# Case Report: Group B *Streptococcus* meningitis in an adolescent****

**DOI:** 10.12688/f1000research.4651.1

**Published:** 2014-07-22

**Authors:** Roselle Vittorino, Joyce Hui-Yuen, Adam J. Ratner, Amy Starr, Teresa McCann

**Affiliations:** 1 Division of Child and Adolescent Health, Department of Pediatrics, Columbia University Medical Center, New York, NY, 10032, USA; 2 Division of Pediatric Rheumatology, Department of Pediatrics, Columbia University Medical Center, New York, NY, 10032, USA; 3 Division of Pediatric Infectious Diseases, Department of Pediatrics, Columbia University Medical Center, New York, NY, 10032, USA

## Abstract

*Streptococcus agalactiae *(group B *Streptococcus*, GBS) usually colonizes the gastrointestinal and lower genital tracts of asymptomatic hosts, yet the incidence of invasive disease is on the rise *. *We describe a case of an 18 year old woman, recently diagnosed with lupus, who reported a spontaneous abortion six weeks prior to her hospitalization.  She presented with fever, altered mental status, and meningeal signs, paired with a positive blood culture for GBS. Magnetic resonance imaging of her brain demonstrated an extra-axial fluid collection, and she was diagnosed with meningitis.  She received prolonged intravenous antibiotic therapy and aggressive treatment for lupus, leading to clinical recovery. This case illustrates the importance of recognizing GBS as a potential pathogen in all patients presenting with CNS infection *.  *

## Introduction

In this case report we describe an interesting and unique patient with group B Streptococcus (GBS) meningitis following a spontaneous abortion without instrumentation. Although our patient was recently diagnosed with systemic lupus erythematosus (SLE), this chronic disease has not been shown to increase the chances of developing invasive GBS infection ^1^. Although GBS can cause severe infections outside of the neonatal period, it rarely manifests as meningitis, accounting for less than 2% of all cases of invasive GBS in the United States and Spain ^2, 3^. There have been only two reported cases of GBS meningitis following elective termination with cervical dilatation and uterine evacuation, both presenting to a hospital in Detroit, Michigan ^4, 5^ and none to date following spontaneous abortion.

## Case report

In October, 2013, an 18-year-old African American young woman diagnosed in August of that year with SLE presented to a community hospital complaining of headache, neck and back pain for five days, and vomiting with non-bloody diarrhea for one day. Additionally, she reported right-sided weakness. Six weeks prior to this presentation, she met the criteria for a diagnosis of SLE with arthritis, a photosensitive malar rash, and serum serology positivity for anti-nuclear antibodies (1:5120) as well as anti-double stranded DNA and anti-ribonuclear protein antibodies. A regimen of oral prednisone (10 mg twice daily) and hydroxychloroquine (200 mg twice daily) was begun. In the weeks subsequent to the diagnosis and treatment of SLE, and prior to her hospitalization, the patient had a positive home pregnancy test, which was followed by an unusually heavy menstrual cycle and presumed spontaneous abortion. The patient did not seek medical attention, and therefore no uterine manipulation or evacuation was performed. The patient’s family history is significant for a maternal aunt and paternal cousin with SLE, and a maternal great-uncle who died from Libman-Sacks endocarditis.

At the time of the acute presentation, she was febrile to 38.9°C, oriented to self only, and had positive Kernig and Brudzinski signs. A computed tomography scan of the head revealed an extra-axial fluid collection in the right frontotemporal region, with a mild local mass effect, but no midline shift and normal ventricles. Intravenous vancomycin and ceftriaxone were initiated and the patient was transferred to our institution.

**Figure 1.**
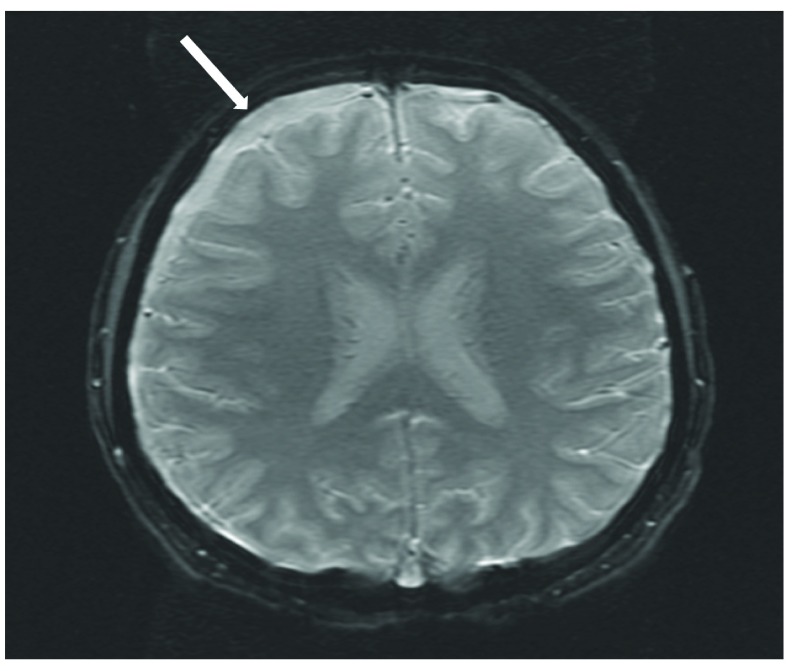
Axial susceptibility weighted image of MRI brain with an arrow indicating the extra-axial collection in right frontotemporal region.

**Figure 2.**
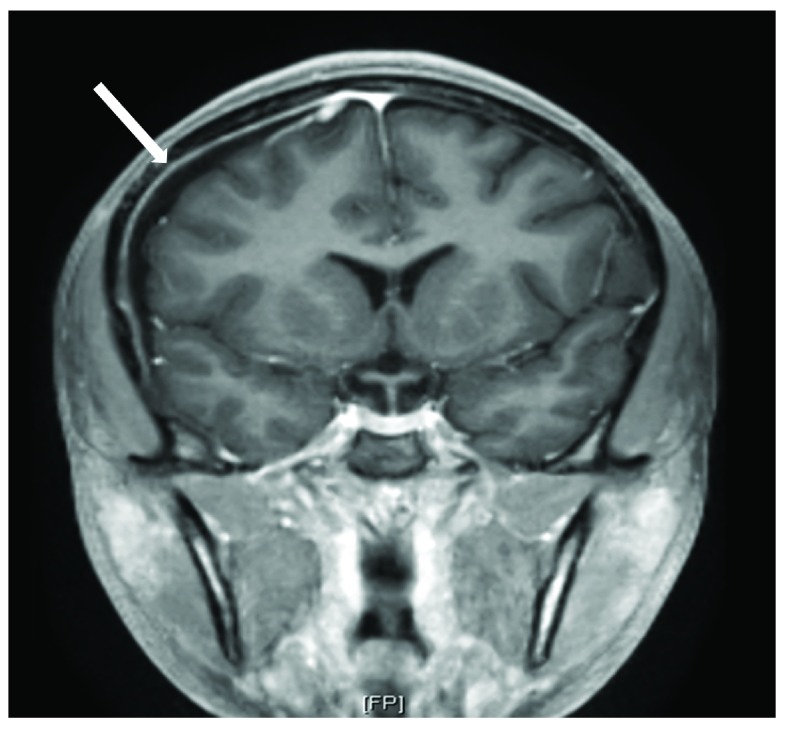
Coronal view of MRI brain post-contrast showing extra-axial collection with the arrow indicating dural enhancement.

On arrival to our pediatric intensive care unit, the patient’s temperature was 38.8°C, blood pressure was 145/76 mmHg, heart rate was 70 beats per minute, respiratory rate was 22 breaths per minute, and oxygen saturation was 100% on room air. She was drowsy and irritable when aroused, complaining of continued severe headache and neck pain. Neurological exam was significant for photophobia and meningismus. The remainder of the neurological exam and the ocular exam were normal. Analysis of the cerebrospinal fluid (CSF) revealed 1525 white blood cells/µL (91% neutrophils), 65 red blood cells/µL, 252 mg/dL protein, and 56 mg/dL glucose. An MRI of the brain with and without contrast revealed a moderately sized extra-axial effusion with secondary inflammatory response ( Figure 1 and Figure 2) and punctate diffusion abnormalities within the dependent portion of the lateral ventricles, consistent with pus or debris. The CSF culture was negative for bacteria, likely secondary to receipt of antibiotics prior to sampling, but the blood culture prior to antibiotic receipt grew GBS. Antibiotic susceptibility testing was not performed. CSF studies for other pathogens, including cryptococcal antigen, detection of herpes simplex and varicella viruses by PCR, and specific cultures for acid-fast bacilli and fungi, were negative. Upon identification of GBS, vancomycin and ceftriaxone were discontinued, and treatment was continued with intravenous penicillin G (4,000,000 units every 4 hours) for two weeks. The patient showed rapid improvement on antibiotic therapy and had resolution of all neurologic symptoms within one week of admission. She was discharged to receive intravenous ceftriaxone for one additional week at home.

Notably, the patient’s SLE was severely active during the course of this acute illness. Initial laboratory results at our institution demonstrated anti-Smith, anti-Ro and anti-La antibodies. She also had severe hypocomplementemia, doubling of serum creatinine, and nephrotic range proteinuria as assessed by 24-hour urine collection. Subsequent kidney biopsy demonstrated class V lupus nephritis. Induction therapy with intravenous methylprednisolone was started, which was then changed to oral prednisone (30 mg twice daily), mycophenolate mofetil (1000 mg twice daily), and hydroxychloroquine (400 mg once daily). Her serologically active SLE began to improve after her course of methylprednisolone, but was still active at the time of discharge.

## Discussion

GBS is an encapsulated bacterium that produces a narrow zone of hemolysis on sheep blood agar. Strains are classified into one of 10 serotypes determined by the organism’s polysaccharide capsule. GBS colonizes the gastrointestinal tract, vagina, and urethra of asymptomatic hosts, but can cause invasive disease in infants, pregnant or post-partum women, individuals with underlying medical conditions and the elderly ^6– 9^.

GBS emerged as the leading cause of neonatal sepsis in the 1970s in the US ^10^. While the rates of early-onset invasive GBS infection among neonates have declined due to widespread use of intra-partum antibiotic prophylaxis in the United States, GBS continues to affect an estimated 1200 infants per year ^9^. Pregnancy and the post-partum state are characterized by an altered immune response that allows GBS to cause invasive disease ^11^. A 6-year epidemiologic study done in the US found that half of all invasive GBS infections seen in pregnant women were associated with infection of the upper genital tract, placenta, and amniotic sac, leading to fetal demise ^12^. Other commonly reported manifestations included bacteremia (31%) and endometritis without fetal death (8%). Deutscher *et al.* reported that a greater proportion of cases of invasive GBS disease occurred in the post-partum period (14% vs. 10% in pregnant women), and pregnant women had a 2-fold increase incidence of GBS disease compared to non-pregnant women ^11^.

As the rate of neonatal GBS infection has declined, the majority of cases now occur in the adult population, manifesting as skin or soft tissue infection, bacteremia, pneumonia, osteomyelitis and septic arthritis ^2, 3, 6, 7, 12^. Several studies have shown increasing rates of invasive GBS infection in all adult age groups, but particularly vulnerable populations are those with medical co-morbidities and the elderly. Of those with underlying medical conditions, 41% have diabetes mellitus, 36% cardiovascular disease, and 17% malignancy ^12^. Interestingly, immunosuppressed individuals comprise less than 10% of all at risk groups ^2, 3^.

GBS meningitis is a rare occurrence, accounting for <2% of all cases of invasive GBS infection in the US and Spain ^2, 3^. In fact, one of the largest initial reviews of GBS meningitis in the adult population identified only 12 cases over a 15 year period, and found 72 cases in the literature overall. The majority of those cases were associated with underlying medical conditions and the risk of contracting GBS meningitis was greater with increasing age, with 58% of cases found in individuals >50 years old ^13^. Although pregnant women have a 2-fold increased incidence of GBS disease compared to non-pregnant women, meningitis caused by GBS remains rare in this population ^14– 18^.

Infection of the central nervous system (CNS) is uncommon among patients with SLE, and relevant symptoms may resemble neuropsychiatric lupus flare, making diagnosis difficult ^1^. There does not appear to be a temporal association between contraction of CNS infection and diagnosis of SLE ^19^, but CNS infection does account for a significant amount of mortality in patients with SLE ^20^. To date, the largest cohort of SLE patients with CNS infections is a group of 38 patients, none of whom had GBS, collected over a 10-year period ^19^. In a large retrospective review of Korean patients with SLE, only 1.4% had meningitis, with the leading causative organism being *Cryptococcus neoformans* ^21^. A recent study characterizing the major infections seen in juvenile onset SLE found meningitis to be a rare entity. Only one case was identified out of 101 major infections, with the causative agent being *Streptococcus pneumoniae* ^22^. Only three other cases of GBS meningitis in patients with SLE have been reported ^3, 23^.

To our knowledge, this patient represents the only reported case of GBS meningitis following a spontaneous abortion without instrumentation of the uterus. The two previously reported cases of GBS meningitis followed elective abortion with cervical dilatation and uterine evacuation ^4, 5^. Although these women became symptomatic within two weeks of their procedure, a recent study of expectant versus surgical management of first-trimester miscarriage showed that 18.6% of women in the expectant management group had evidence of retained products of conception after four weeks ^24^. Therefore it is possible that our patient remained at risk for invasive infection longer than would otherwise be expected.

This case illustrates the importance of recognizing GBS as a potential pathogen in all patients presenting with CNS infection. It also highlights the value of obtaining a thorough obstetrical history in all reproductive-aged females, even in the setting of acute illness.

## Consent

Written informed consent for publication of the patient’s clinical details and images was obtained from the patient.
