# Supplementary material for: Case Report: Group B Streptococcus meningitis in an adolescent  
Source: F1000Res. 2014 Jul 22;3:167. [Version 1] doi: 10.12688/f1000research.4651.1 (PMC4193390; doi:10.12688/f1000research.4651.1)
Supplement: Peer review history

# Peer review: Referee response for version 1

**DOI:** 10.5256/f1000research.4972.r5871

**Published:** 2014-10-10

**Authors:** Rebecca Madan

**Affiliations:** 1 Department of Pediatrics, Yeshiva University, Bronx, USA

This case report reviews an unusual and thought-provoking case of Group B Streptococcus meningitis in a young woman who had a recent history of both spontaneous abortion and newly diagnosed SLE. Of note, she received relatively low-dose immunosuppression prior to developing meningitis, and the authors indicate that SLE participants are typically not thought to be at higher risk for bacterial meningitis.  This case report is highly relevant in that it illustrates the imperative to 1) exclude infection in the setting of altered mental status in SLE patients and 2) to investigate for potential sources of infection (recent pregnancy) in a patient with an invasive bacterial infection that is not typical of their age group.

The authors imply in their Discussion that this case of GBS meningitis was more likely to be related to the patient's recent history of spontaneous abortion and was less likely related to her recently diagnosed SLE. They cite a significant prevalence of endometritis (8%) in women who develop invasive GBS with pregnancy. Did the patient have evidence of endometritis on exam or imaging? Did she undergo pelvic imaging to assess for retained products of conception (or undisclosed foreign body) that could provide an ongoing source of infection?

The most interesting aspect of this case is this patient's significant SLE flare in the setting of GBS meningitis. The authors report that she had serologic evidence of SLE flare, hypocomplementemia, and a new diagnosis of class V lupus nephritis concomitant with her GBS diagnosis. This presents an interesting finding to expand upon in the Discussion. Although bacterial meningitis is unusual in the setting of SLE, is it possible that this young woman developed a severe flare prior to meningitis, and that the subsequent immune dysfunction predisposed her to invasive GBS? Or do the authors postulate that immune dysfunction in the setting of bacterial sepsis resulted in the SLE flare? What are the possible mechanisms that could underly a relationship between these two conditions? A brief discussion of the patient's SLE flare would contribute significantly to this manuscript.

I have read this submission. I believe that I have an appropriate level of expertise to confirm that it is of an acceptable scientific standard.

# Peer review: Referee response for version 1

**DOI:** 10.5256/f1000research.4972.r5546

**Published:** 2014-08-04

**Authors:** Theresa Lu

**Affiliations:** 1 Department of Microbiology and Immunology, Weill Cornell Medical Center, New York, NY, USA

This is a well-written case report about a newly diagnosed lupus patient who develops GBS meningitis after a spontaneous abortion.  Most of the salient details are included and there is an informative review of the literature on GBS infections, autoimmune disease, and spontaneous abortions.  This is a useful documentation of an unusual case that can be helpful when considering the differential diagnosis of meningitis in a patient with lupus, other autoimmune disorders, or spontaneous abortion.

One presumes that the patient was in her first trimester, but confirmation of that in the description of the patient would be helpful to be complete.

I have read this submission. I believe that I have an appropriate level of expertise to confirm that it is of an acceptable scientific standard.
